# On-screen fingerprint sensor with optically and electrically tailored transparent electrode patterns for use on high-resolution mobile displays

**DOI:** 10.1038/s41378-020-00203-4

**Published:** 2020-11-02

**Authors:** Hyun-Joon Kim-Lee, Seog Woo Hong, Dong Kyun Kim, Jinmyoung Kim, Hong Suk Kim, Seok-Whan Chung, Eun-Hyoung Cho, Hae-Sung Kim, Byung-Kyu Lee

**Affiliations:** Device Research Center, Samsung Advanced Institute of Technology, 130 Samsung-ro, Yeongtong-gu, Suwon-si, Gyeonggi-do 443–803 Republic of Korea

**Keywords:** Electrical and electronic engineering, Micro-optics

## Abstract

In this study, a mutual capacitive-type on-screen fingerprint sensor, which can recognize fingerprints on a display screen to provide smartphones with full-screen displays with a minimal bezel area, is fabricated. On-screen fingerprint sensors are fabricated using an indium tin oxide transparent conductor with a sheet resistance of ~10 Ω/sq. and a transmittance of ~94% (~86% with the substrate effect) in the visible wavelength range, and assembled onto a display panel. Even at this high transmittance, the electrodes can degrade the display quality when they are placed on the display. The interference between periodic display pixel arrays and sensor patterns can lead to the Moiré phenomenon. It is necessary to find an appropriate sensor pattern that minimizes the Moiré pattern, while maintaining the signal sensitivity. To search for appropriate patterns, a numerical calculation is carried out over wide ranges of pitches and rotation angles. The range is narrowed for an experimental evaluation, which is used to finally determine the sensor design. As the selected sensor pitches are too small to detect capacitance variations, three unit patterns are electrically connected to obtain a unit block generating a larger signal. By applying the selected sensor pattern and circuit driving by block, fingerprint sensing on a display is demonstrated with a prototype built on a commercial smartphone.

## Biometrics: Creating transparent cell phone fingerprint scanners

Capacitive fingerprint sensor modules have been developed that avoid visual clashing with the pixels of cell phone screens. Capacitive fingerprint modules profile how individual fingerprints change their ability to store small electrical charges. As their electrodes and cell screen pixels share similar intervals, a visual distraction known as a Moiré pattern arises when the two are superimposed. Through calculations and experimentation, Hyun-Joon Kim-Lee and a team from the Samsung Advanced Institute of Technology in South Korea assayed a variety of electrode intervals and rotations to find the combination with the least visual interference. The chosen electrode pattern interval was initially too small for fingerprint detection, an obstacle the team overcame by making groups of electrodes send and receive signals together. Further studies could bring capacitive fingerprint sensors to flexible devices or produce combination touch sensors.

## Introduction

Full-screen smartphones with extremely thin bezels without physical buttons on the front have become the mainstream in the smartphone industry in recent years. For this type of smartphone, fingerprint sensors, formerly located under the home button, have to be positioned on the back or side of the smartphone. However, the most convenient and intuitive location of the fingerprint sensor is on the front face. Various types of sensors that can recognize fingerprints on display screens have been developed^1–11^. Typical examples include capacitive, optical, and ultrasonic fingerprint sensors. Among them, optical and ultrasonic sensors are used in some smartphone models because they can be placed under the display and do not interfere with display imaging, even though they are relatively bulky and costly. However, in some cases, these sensors may not work as satisfactorily as conventional fingerprint sensors. The optical under-display fingerprint sensor, which recognizes a fingerprint using the difference in light reflected at the finger ridge and valley areas, has an issue in recognizing dry fingers, which cannot make uniform and consistent contact with the surface of the sensor cover layer^12,13^. This issue arises because the sensor may identify a finger ridge area, which is not making good surface contact, as a valley area since part of the light will be reflected at the cover surface instead of propagating through the finger on the surface. Ultrasonic under-display fingerprint sensors also have room for improvement in terms of recognition time, dry finger recognition, and manufacturing yield^10^. The mutual capacitive-type on-screen fingerprint sensor is another promising candidate because its not-on-screen version has been employed in a large number of commercial smartphones, and its performance has been confirmed. Mutual-capacitive-type on-screen fingerprint sensors can be integrated with touch sensors in the same substrate layer, and fabricated on a large area (>200 mm^2^) without considerable additional cost. In addition, these sensors can work on both OLED (organic light-emitting diodes) and LCD (liquid crystal display) screens, while optical and ultrasonic on-screen sensors can work with OLED displays only. In addition, as these sensors can be easily fabricated on a polymer film substrate, they are better suited for foldable smartphones compared to other types of sensors. Therefore, mutual capacitive-type on-screen fingerprint sensors are being extensively developed. The design and material issues regarding the employment of such a sensor are presented in this paper.

**Fig. 1:**
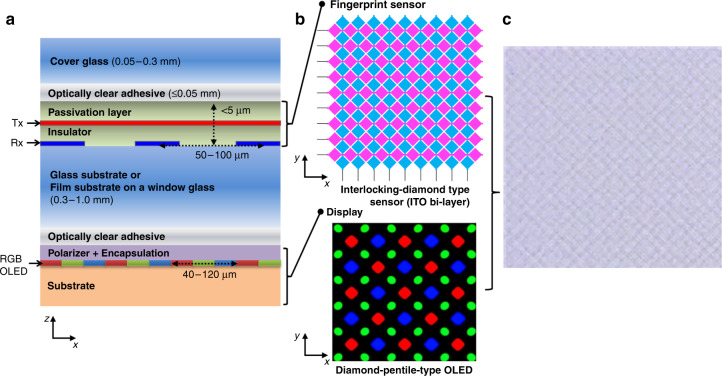
Schematic of Moiré pattern generation due to the on-screen fingerprint sensor. **a** Stack structure of the capacitive on-screen fingerprint sensor. **b** Top views of typical fingerprint sensor (ITO interlocking diamond type) and OLED display patterns. **c** Moiré pattern generated by the overlap of the ITO fingerprint sensor with a pitch of 70 μm and the diamond-pentile-type 570 ppi OLED display

The mutual capacitive-type on-screen fingerprint sensor stack structure is shown in Fig. 1a. As the mutual capacitive-type fingerprint sensor has a limited sensing distance, typically ≤300 μm (refs. ^14,15^), it needs to be on top of the display panel and covered with a thin cover glass. The sensor can be fabricated on a glass substrate or a polymer film bonded on a window glass. In either case, a relatively thick glass layer (0.3–1 mm) is required between the display panel and sensor to make the cover glass and overall structure mechanically robust and minimize the display noise. As the cover glass and thick lower glass are bonded with a thin polymer film and an adhesive layer in between, the overall structure can behave as a strong laminated glass. The operation principle of the sensor is the same as that of the general home button fingerprint sensor. However, on-screen sensors should have transparent electrodes to not block or distort display images. As the sensing electrodes are “on” the display and the “transparent” electrodes are not completely transparent, the greatest concern in the application of this type of sensor is the possible degradation of the display image quality. Specifically, the appearance of Moiré patterns can be very problematic. The Moiré pattern refers to a phenomenon in which a new pattern, which did not originally exist, becomes apparent when repeated patterns or structures, such as grids, meshes, and a pixel matrix, overlap^16–18^. In the case of the on-screen fingerprint sensor, a repetitive pattern of the display overlaps with another repetitive pattern of the sensor. Consequently, a Moiré pattern is observed, which can significantly reduce the display image visibility^5^.

The Moiré pattern issue has been extensively studied for the substitution of indium tin oxide (ITO) with metal mesh electrodes in touch screen panels, as they also have sensor electrodes on the display panel^17–19^. In the touch sensor, the pitch of the electrodes is considerably larger than that in the fingerprint sensor (~4 mm vs. ~50–100 μm)^20,21^, so it can only locate the finger as a whole. The pitch is considerably larger than the pitch of smartphone display pixel arrays (~40–120 μm); thus, it is less challenging to avoid or reduce the Moiré pattern by altering the pattern geometry without significantly affecting the sensing capability^17,19,22^. However, the pitches of the fingerprint sensor electrodes are in the same range as the smartphone display pixel pitch, and thus, the Moiré effect can be significant. For example, the Samsung Galaxy S10 display has a pixel pitch of 46 μm (550 ppi), while a commercial fingerprint sensor has a pitch of ~50–85 μm (300–500 ppi). In addition, the electrodes must have a very low sheet resistance (~10 Ω/sq.) compared to those of touch sensors (>100 Ω/sq.), as the fingerprint sensor needs to be driven at a higher frequency, which leads to a lower transparency (~86% with the substrate effect without patterning) and a Moiré pattern with a higher contrast^23,24^. If a conventional interlocking diamond electrode pattern (Fig. 1b) overlaps the display without rotation, a Moiré pattern (Fig. 1c) appears. This Moiré pattern issue needs to be overcome to implement the on-screen fingerprint sensor in smartphones. In this study, the pitches and rotation angles of the diamond-type ITO electrode patterns that can minimize the Moiré phenomenon on the smartphone display panel are searched through various simulations and experimental observations. The expected electrical properties of selected sensor patterns are calculated to evaluate whether they can have sufficient sensitivities for fingerprint authentication. Sensors with the selected pattern design are fabricated and integrated into prototypes to demonstrate the fingerprint image acquisition on a smartphone display.

## Results and discussion

### Sensor pattern selection for Moiré pattern minimization

**Fig. 2:**
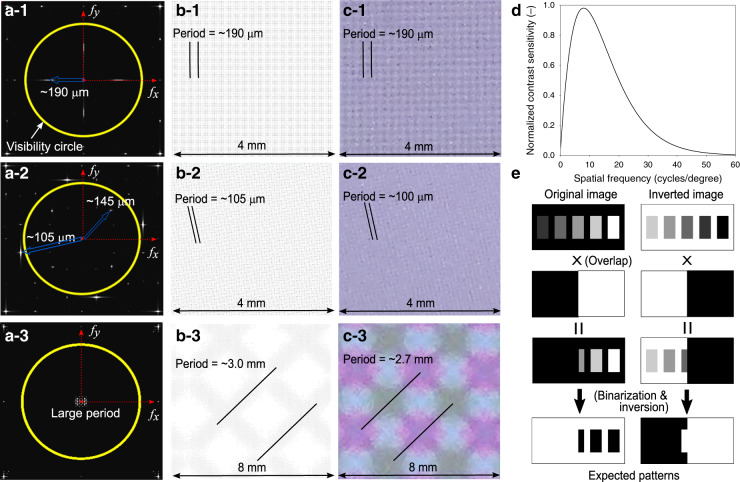
Numerical and experimental Moiré analysis results for selected sensor patterns. **a** DFT calculation results, **b** approximate real-space pattern extraction images, and **c** observed Moiré patterns for the sensors with **1** a pattern pitch of 20 μm and a rotation angle of 0°, **2** a pattern pitch of 25 μm and a rotation angle of 38°, and **3** a pattern pitch of 45 μm and a rotation angle of 0° on a 570 ppi smartphone display. **d** Human CSF^28^. **e** Binary Moiré pattern extraction scheme

**Fig. 3:**
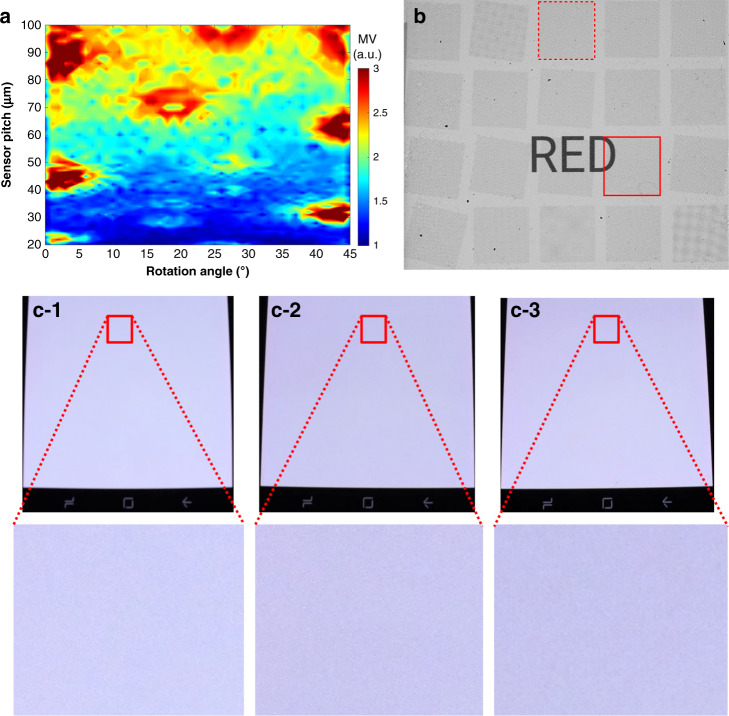
Sensor pattern selection and observation on a smartphone screen. **a** MV plots calculated for various sensor pitch and rotation angle combinations. **b** High-resolution camera images showing the Moiré patterns from the 20 candidate sensor patterns. Smartphone screens (**c-1**) without a fingerprint sensor pattern and (**c-2**, **c-3**) with fingerprint sensor patterns on them (**c-2**: a pattern with a pitch of 25 μm and a rotation angle of 38°, **c-3**: a pattern with a pitch of 20 μm and a rotation angle of 8°)

Considering the nature of the on-screen fingerprint sensor, which must acquire signals at regular intervals on a digital display device, Moiré patterns cannot be avoided. Therefore, the Moiré pattern should be made imperceptible through a proper combination of sensor geometrical parameters. A spectral analysis of overlapped display–sensor pattern pairs in the frequency domain can be carried out by Fourier transform to predict the Moiré pattern periods, directions, and strengths, i.e., perceptual contrast^16–18,25,26^. Therefore, in this study, grayscale *N* × *N* pixel images of various overlapped patterns were prepared, and the discrete Fourier transform (DFT) was applied to the image matrices to perform spectral analysis. When the image is expressed as the gray-level intensity distribution function *f(x,y)*, where *x* = 0, 1, 2, …, *N* − 1 and *y* = 0, 1, 2, …, *N* − 1, it can be Fourier transformed into

1 $$F\left( {u,v} \right) = \mathop {\sum }\limits_{x = 0}^{N - 1} \mathop {\sum }\limits_{y = 0}^{N - 1} f\left( {x,y} \right){\mathrm{exp}}\left[ { - \frac{{2\pi j(ux + vy)}}{N}} \right],$$

where *u* and *v* are the horizontal and vertical components of the frequency vector, respectively. *F(u,v)* provides the amplitude, which corresponds to the perceptual contrast of the Moiré pattern, at the spatial frequency represented by *u* and *v*, and can be plotted as in Fig. 2a (ref. ^25^). The white or gray dots in the plot, indicated with blue arrows in Fig. 2a–1, a–2 and clearly shown around the origin in Fig. 2 a–3, denote the spatial frequency vector of the patterns in the overlapped images. The amplitude is represented by the brightness of the dots. The yellow circle in Fig. 2a represents the visibility circle beyond that fine details of the pattern cannot be detected by the human eye at a viewing distance of 40 cm. The viewing distance was assumed to be ~40 cm, as the mean eye–screen distance is ~33–40 cm for mobile devices^27^. The radius of the visibility circle corresponds to the threshold spatial frequency, 60 cycles/degree, suggested by the human contrast sensitivity function (CSF)^17,18^. The CSF describes the threshold sensitivity of the visual system to sinusoidal luminance contrast, with a range of spatial frequencies. Although various forms of the function have been reported, most CSFs become 0 if the spatial frequency is >60 cycles/degree. A typical CSF is

2 $$C_S\left( {f_S} \right) = \left[ {0.0499 + 0.2964\,f_S} \right] {\mathrm{exp}}\left[ { - (0.114\,f_S)^{1.1}} \right],$$

where *f*_*S*_ is the spatial frequency of the visual stimuli in cycles per degree. The function is normalized to a maximum value of 1, as shown in Fig. 2d (ref. ^28^). This equation indicates that a pattern with a very low contrast can be observed if the spatial frequency of the pattern is ~8 cycles/degree, where the contrast sensitivity is highest. In addition, patterns will not be noticeable when the spatial frequency is >60 cycles/degree, regardless of the contrast level because human eyes are not sensitive to patterns with these large spatial frequencies.

In addition, approximate Moiré patterns were obtained by extracting bright and dark areas of overlapped two-dimensional (2D) images directly in the image domain as a complementary approach to the spectrum analysis. We extracted the expected Moiré patterns by converting the overlapped grayscale images and their inversions into binary images, as simply described in Fig. 2e. As the binarization simplifies the pattern and enhances the contrast, patterns can be easily recognized from at least one of the two binary images, either the original or the inverse, and compared with the observed results.

These analysis methods were used to predict Moiré patterns for the selected smartphone display (diamond-pentile-type OLED, 570 ppi), and diamond-type ITO sensors with electrode pitches of 20–100 μm at an interval of 1 μm and rotation angles of 0–45° every 1°. Owing to the fourfold symmetry (Fig. 1b), the angle was varied from 0 to 45°. DFT calculations and pattern extraction were performed with overlap of a fixed display pattern image and rotated sensor pattern images in grayscale. Some of the analysis results are shown in Fig. 2a, b. Figure 2a–1 shows the DFT calculation result for the sensor pattern with a pitch of 20 μm without rotation. Inside the visibility circle, four manifest white dots are observed, which denote the frequency vectors of the main Moiré pattern. The directions and periods of the Moiré patterns in real space can be obtained by using the frequency vectors. The directions and periods coincide with those of the extracted real-space pattern in Fig. 2b–1, and the pattern in Fig. 2c–1 obtained by the experimental evaluation presented in the next section. Notably, Fig. 2a–2 predicts more than one pattern period. However, the peaks just outside the visibility circle are considerably higher than those inside the circle, and the pattern corresponding to the outside peaks is dominant, as shown in Fig. 2b–2, c–2. In Fig. 2a–3, the expected Moiré pattern frequency vectors are very close to the origin, thereby suggesting a very noticeable Moiré pattern with a period of several millimeters, as shown in Fig. 2b–3, c–3.

Considering the large number of different pitch and rotation angle combinations, a criterion that can quantify the visibility of the Moiré pattern is required. Therefore, in this study, approximate Moiré visibility (MV) values for each pitch and rotation angle were calculated for comparison and selection of the patterns,

3 $${\mathrm{MV}} = \mathop {\sum }\limits_{u = 0}^{N - 1} \mathop {\sum }\limits_{v = 0}^{N - 1} F\left( {u,v} \right) C_S\left( {\sqrt {u^2 + v^2} } \right).$$

This value quantitatively reflects the perceptual contrast of the pattern filtered with the CSF in the frequency domain. However, this value needs to be used carefully to narrow the search range, instead of finding the minimum and selecting the optimal pattern, as the simple summation can be misleading. For example, the values can be similar in the cases of one very bright dot in the spectral analysis plot and of many less bright dots, although the Moiré pattern can be more manifest in the former case. Nevertheless, the MV values significantly vary with changes in the sensor pattern and can be effectively used to narrow our selection of patterns for the next step. The pattern contrast in the image domain can be quantified after inverse Fourier transform of the MV, although the inverse Fourier transform results have limitations similar to those of the MV values^17,18^. Nevertheless, 20 candidate patterns were selected based on the MV calculation results shown in Fig. 3a. The results show that the smallest MV values can be found in the 30 µm pitch case around the rotation angles of 7–12 degrees, 15–20 degrees, and 35–40 degrees. Therefore, most of the candidates were selected from the cases, where the sensor pattern pitch is 30 µm or smaller.

After the analysis described above, 55 patterns were fabricated on a glass wafer to obtain an area of 10 mm × 10 mm for each pattern. The other 35 test patterns in addition to the 20 candidate patterns mostly had sensor pitches of ~60–70 µm, which are commonly used in conventional mutual-capacitive fingerprint sensors, and have been verified for sensing capability. The goal was to determine whether a certain angle of rotation could make the sensors with these pitch ranges unexpectedly have minimized Moiré patterns, but the results were negative. The patterns were created using the same process used to fabricate the sensor, except that the electrical connections and pads were omitted. Therefore, the patterns consisted of lower ITO, organic insulator, upper ITO, and organic passivation layers. After fabrication, the glass wafer was diced to obtain the same size as that of the Samsung Galaxy S8 display panel. The diced glass substrate was placed on the smartphone’s full-white-mode OLED with glycerine in between. The generated patterns were carefully observed and evaluated. In addition, grayscale photographic images were acquired in a dark environment with a high-resolution industrial camera used for display panel inspection, as shown in Fig. 3b. The images were carefully acquired to avoid additional Moiré or other artifacts owing to the camera and for consistency with the naked eye observations. Although no standard method has been reported for the quantification of a Moiré pattern, the contrasts of different images of Moiré patterns can be compared with the standard deviations of the image pixels’ gray-level values, and the number of sensor pattern candidates can be reduced. In addition, mean opinion scores, popular indicators of the perceived medium quality^29^, were obtained for 10 preselected candidates from 12 adults to choose the best sensor pattern that does not degrade the display quality. A pattern with a pitch of 20 μm and a rotation angle of 8° (in a dashed square in Fig. 3b), and a pattern with a pitch of 25 μm and a rotation angle of 38° (in a dashed square in Fig. 3b) were selected. Larger evaluation samples of these two patterns were generated to cover the whole front side of the smartphone display and compared to the case without the sensor pattern, as shown in Fig. 3c. Except for slight changes in color and brightness, no noticeable Moiré pattern was observed. In addition, no significant difference was observed between the display without a sensor pattern and the display with a sensor on it.

### Sensing signal calculation and sensor pattern selection

**Fig. 4:**
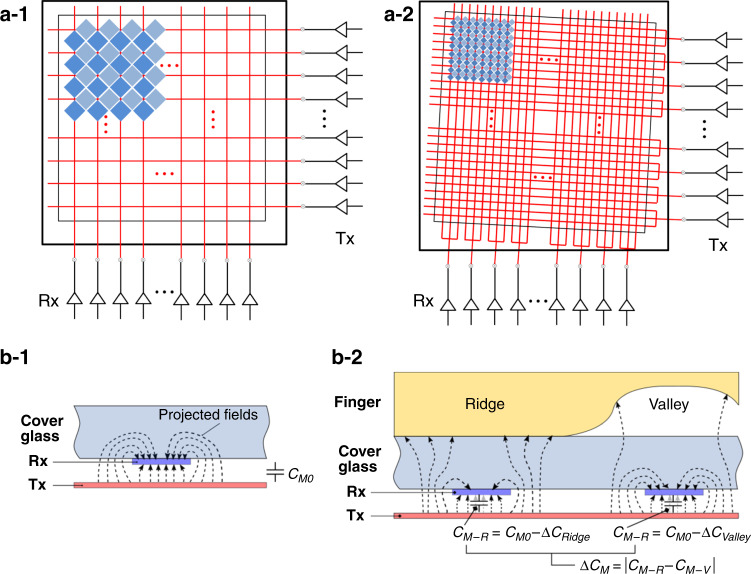
Electrode patterns and sensing principle. **a**–**1** Conventional electrode patterns and sensor driving configuration. **a**–**2** Conceptual rotated electrode patterns with a small pitch, and corresponding sensor driving configuration used in this study. **b**–**1** Mutual capacitance (*C*_M0_) without a finger on the cover glass. **b**–**2** Change in capacitance upon finger contact, and mutual capacitance values at the finger ridge and valley areas

The dimensions of the selected electrode pattern based on the Moiré evaluation are considerably smaller than those of commercial capacitive fingerprint sensor patterns (in the range of 50–100 μm). Thus, the signal-to-noise ratio of the sensor could be significantly decreased, and the sensor might not operate properly. Therefore, a method that can provide a sensor with a pitch sufficiently small optically, but sufficiently large electrically to recognize a fingerprint is required. This can be achieved by connecting several electrode patterns as one unit block and driving the sensor by each block, as shown in Fig. 4b. In this manner, for example, an array of a pattern with a pitch of 25 μm will have a sensing pitch of 75 μm by connecting and controlling three electrodes together on both the signal transmitter (Tx) and receiver (Rx) sides. In other words, a 3 × 3 array of a diamond pattern with a pitch of 25 μm can form a unit sensing block.

The validity of this idea was analyzed by calculating the electrical characteristics, such as the capacitance and resistance, for different sensor patterns by COMSOL Multiphysics® simulations. The Tx and Rx electrode structures in the simulations were interlocking diamond patterns of ITO with a sheet resistance of 11 Ω/sq. In addition, the thickness of the insulating layer between the Tx and Rx layers was assumed to be 1 μm, while the gap between diamonds was set to 8 μm, regardless of the pattern pitch owing to our fabrication capability. First, the mutual capacitances, which are the capacitances between the Tx and Rx electrodes forming each sensing node, were calculated for different cases. When no finger touches the cover surface, the mutual capacitance has the baseline value *C*_M0_, as shown in Fig. 4b–1. Once a finger is placed on the cover surface, the mutual capacitance values under the ridge and valley regions of the finger are reduced to *C*_M–R_ (=*C*_M0_ – Δ*C*_RIDGE_) and *C*_M–V_ (=*C*_M0_ – Δ*C*_VALLEY_), respectively. This occurs as the capacitors under the finger lose charge and the capacitance decreases, but the capacitance changes differ between the finger ridge and valley regions owing to the difference in the distance from the electrodes to the finger surface, as illustrated in Fig. 4b–2. In addition, the Tx and Rx electrode linear resistances (*R*_Tx_ and *R*_Rx_) were obtained by the simulations.

Table 1 shows the calculation results for several different cases where the sensor patterns are composed of different numbers of arrays, while the fingerprint sensing pixel pitches are in the reasonable range of 70–85 μm, which correspond to resolutions of 300–363 dpi. Fingerprint images in this resolution range can be effectively used in commercial devices employing a pattern-based matching algorithm^12,21^. The difference between *C*_M–R_ and *C*_M–V_, denoted Δ*C*_M_, is shown in the table instead of the capacitance values because the amplitude of the sensor output signal is determined by Δ*C*_M_. The rotation angles of the sensor patterns are not indicated because they do not considerably affect the electrical characteristics. The baseline mutual capacitances, *C*_M0_, of the unit sensing patterns composed of *n* × *n* arrays increase almost linearly with *n* if the sensing pixel pitches do not significantly differ. The Δ*C*_M_ values do not substantially change with the pattern pitch for the analyzed sensor patterns, which implies that the signal levels of the selected sensor patterns with pitches of 20 or 25 μm are approximately the same as that of the standard sensor with a pitch of 70 μm, which is used for a conventional fingerprint sensor under the home button of smartphones. Although larger *C*_M0_ values can increase the noise level, all of the values in Table 1 are within the range covered by our integrated chip (IC) for fingerprint sensing. The IC for sensor driving and readout was revised from the commercial touch sensing IC to detect a considerably smaller mutual capacitance difference, while maintaining most of the scheme to filter various noise signals^30^.

**Table 1:** Electrical characteristics of various unit sensing patterns calculated by using COMSOL Multiphysics software

Sensor pattern	Sensing pixel pitch (µm)	Sensing pixel density (ppi)	*C*_M0_ (fF)	Δ*C*_M_ for average female finger^a^ (fF)	Δ*C*_M_ for average male finger^a^ (fF)	*R*_TX_ (Ω/10 µml)
1 × 1 array (pitch: 70 µm)	70	363	10.461	0.136	0.169	7.410
2 × 2 array (pitch: 42 µm)	84	303	23.288	0.199	0.226	5.178
3 × 3 array (pitch: 25 µm)	75	340	31.173	0.143	0.164	4.577
4 × 4 array (pitch: 20 µm)	80	319	46.835	0.159	0.163	3.301

^a^The average pitch values between the ridges of male and female fingerprints were calculated by using the numbers of ridges per centimeter reported previously^37^. The obtained values (male: 480 μm, female: 430 μm) were used to calculate △*C*_M0_

Our final sensor pattern selection was carried out based on the resistance–capacitance (RC) time constants of the patterns, as they affect the signal acquisition time. The RC time constant can be estimated by using *C*_M0_ and *R*_Tx_ in Table 1. Although the resistance of the trace also needs to be considered to obtain the overall resistance (*R*) from the IC to each sensing node, the traces consist of metal with a very low resistivity and thus do not significantly affect the overall trend of *R*. The RC constants are expected to increase with decreasing pitch. Accordingly, the array size *n* in a unit sensing block increases, so *C*_M0_ rapidly increases. Although the line resistance of the electrodes per 10 μm, *R*_Tx_, decreases with decreasing pitch owing to the more electrode lines in the unit sensing block, the decrease in *R*_Tx_ is relatively gradual and cannot change the trend of the RC increase. The time for fingerprint image capture in the 4 × 4 array of the pattern with a pitch of 20 μm with the largest RC time constant can be longer than 200 ms, the conventional limit for fingerprint sensors, at a typical driving frequency of 1 MHz with repetitive signal integration to obtain a sufficiently good fingerprint image. Therefore, the 20 μm 4 × 4 array pattern was excluded to finally select the 25 μm 3 × 3 array with a rotation angle of 38° as our sensor pattern.

### Characterization and demonstration of fingerprint sensors

**Fig. 5:**
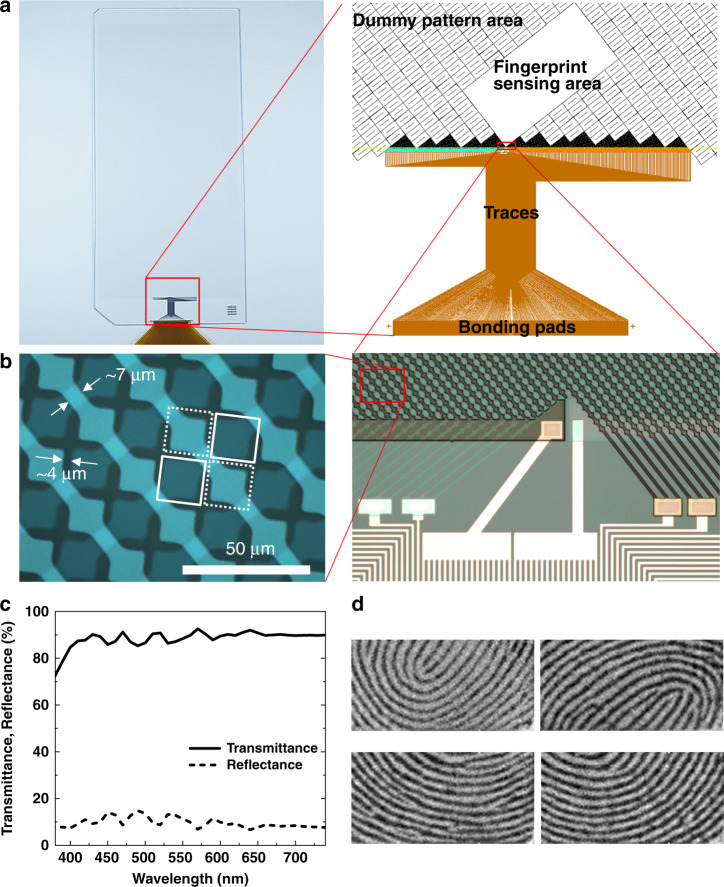
Characterization of the proposed fingerprint sensor. **a** (Left) Transparent fingerprint sensor fabricated on a glass substrate, and (right) schematic of sensing and dummy pattern areas and metal interconnects (i.e., traces) connecting the sensor electrodes to the pads for bonding to the FPCB. **b** Optical microscopy images of (left) part of the sensor pattern area, and (right) a lower section of the sensor pattern and vias connected to the traces. White squares drawn with solid lines and dotted lines indicate lower and upper ITO diamond patterns, respectively. The upper ITO electrodes appear bright in the left image, but are relatively dark in the right image owing to the different lighting and organic coating conditions. **c** Transmittance and reflectance in the visible wavelength range measured on the sensing area of the fabricated sensor. **d** Fingerprint images obtained by the sensor

Using the selected sensor pattern design, fingerprint sensors were fabricated on glass wafers, and their electrical and optical properties were characterized. The sensors can also be fabricated on the large glass substrates used for the manufacturing of display panels and can be transferred to plastic film substrates if needed. A schematic of the sensor design and optical microscopy images of the fabricated sensor are shown in Fig. 5a, b. The electrodes have smaller diamond shapes with thick and long necks between them compared to conventional touch sensors or fingerprint sensors, as the width of the necks and spacing between adjacent upper and lower diamonds cannot be reduced in proportion to the pitch of the diamond pattern. The width of the necks was maintained at 7 μm to ensure proper resistance levels of the electrodes, while the spacing was maintained at 4 μm to secure the alignment margins. The right image in Fig. 5b shows that three electrode lines are connected to one via, and that one interconnect line exits from the via, as mentioned above.

Considering the literature results and preliminary experiments, we targeted an ITO electrode thickness of 200 nm to obtain proper combinations of sheet resistance and transmittance^23,24^. ITO was selected as an electrode material for the sensor owing to its reasonable resistance and transmittance along with its well-established mass manufacturing technologies, although other types of transparent conductors, such as oxide–metal–oxide or metallic nanowires/nanostructures, were also considered^31–34^. The sheet resistance of the fingerprint sensor’s ITO measured using the electrical test element group pattern fabricated with the sensor was in the range of 10–12 Ω/sq., as expected, sufficiently low for sensor driving and consistent with the value used for the simulations. The sensor optical properties were measured using a spectrophotometer, while the electrodes were covered with an organic passivation layer without cover glass. The measured transmittance and haze in the visible light range were 88–89% and 2.5%, respectively, with the substrate effect, as shown in Fig. 5c. The transmittance is slightly higher than that of the unpatterned ITO layer, as the sensors have gaps between the ITO diamond patterns, as shown in the optical microscopy image in Fig. 5b. The use of proper index-matching layers and coating on the cover glass is expected to improve the transmittance and haze close to the desired values (>90% and <1%, respectively). The transmittance of the electrode material is an important factor with respect to the Moiré issue, as well as the brightness of the display and battery life of the smart device. A higher transmittance implies a lower contrast when the patterns overlap, and thus, a pattern having the same period would be less noticeable according to the CSF. Therefore, the Moiré pattern may not be visible when the transmittance of the electrode is very high, even if the electrode pattern is not rotated or the electrode pitch is not adjusted, as suggested in a previous report^5^. However, the contrast sensitivity significantly varies according to the luminance and viewing angle of the object along with other factors^35^. The high-transmittance strategy may not be suitable for some use cases of smart devices. Therefore, in terms of the Moiré pattern, it is better to find and use the sensor pattern with a spatial frequency exceeding the threshold, as in this study.

The fabricated sensor with 64 Tx and 128 Rx channels was connected to an IC controller through a flexible printed circuit board (FPCB). Fingerprint images were obtained on a cover glass attached to the sensor with an optically clear adhesive (OCA). The fingerprint sensor authentication rate was evaluated by using our pattern-based matching algorithm and over 10,000 fingerprint images^36^. The authentication rate was >97% (false rejection rate <3%) at a false acceptance rate of 1/50,000, which is equivalent to the authentication rate of the home-button-type fingerprint sensor currently on the market^21^. Some of the fingerprint images obtained by the sensor are shown in Fig. 5d. A high contrast is observed between the ridges and valleys of the fingerprint. In addition, features such as ridge endings and bifurcations are clearly visible. Approximately four to five sites of features are obtained in the fingerprint images from the 9.6 mm × 4.8 mm sensing area.

**Fig. 6:**
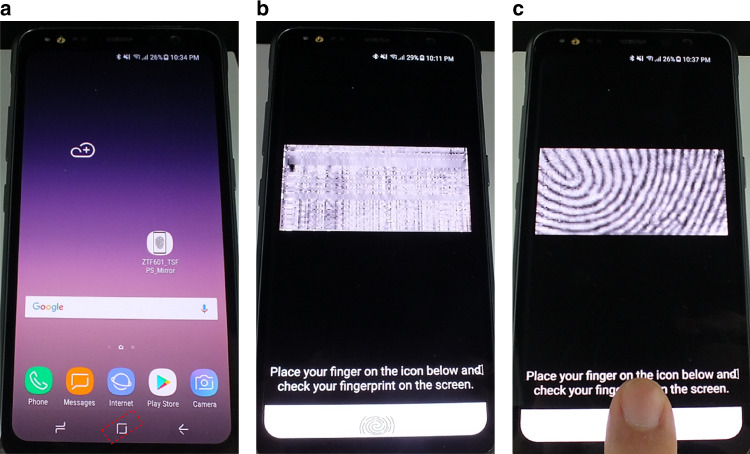
High-resolution smartphone display with an on-screen fingerprint sensor. **a** The sensing area is outlined by the red dashed slanted rectangle in the soft home button area, while the display shows the normal home screen. The fingerprint sensing demonstration app shows the **b** white noise in the absence of objects on the sensor and **c** fingerprint image when a finger is placed on the sensor

Finally, the sensor modules were assembled into smartphone prototypes. The Samsung Galaxy S8 Active’s window glass was replaced with the sensor wafer, which was diced to have the same dimensions as those of the original window glass and laminated with a cover glass. After the assembly, the sensing area is located at the bottom center of the front surface, where the soft home button is displayed. The remaining area is covered with dummy patterns to make the sensing area indistinguishable. Figure 6a shows a functioning smartphone display with the fingerprint sensor laminated on it. Notably, no reduction in image quality in the whole display area is observed. In addition, the location or size of the fingerprint sensor cannot be identified if they are not clearly indicated, such as by the red dashed slanted rectangle in Fig. 6a. A simple android app for fingerprint sensing demonstration was developed and installed on the prototype device to show enlarged images of the recognized fingerprints on the smartphone display. Clear fingerprint images can be obtained from the sensor on the fully functioning display screen of the smartphone, as shown in Fig. 6b, c. The fingerprint image in Fig. 6c is brighter and has a better contrast compared to the images in Fig. 5d, as real-time image enhancement was applied for demonstration purposes. To the best of our knowledge, this is the first study demonstrating a capacitive-type on-screen fingerprint sensor functioning, while integrated in a smartphone display, not just separately.

## Conclusion

The Moiré patterns arising from the fingerprint sensor on a smartphone display screen were evaluated numerically and experimentally to find sensor patterns that minimize the reduction of the display quality. Although the derived sensor pattern pitch was outside of the conventional range for a fingerprint sensor, the simulations showed that the overall sensing capability could be preserved by grouping every few adjacent electrodes such that the adjacent electrodes in each group send and receive signals together. The ITO thin film processing conditions for the sensor that provide the electrodes with the proper combination of transmittance and sheet resistance were determined, and sensors were fabricated under these conditions on glass wafers. Finally, the fabricated fingerprint sensor was integrated into a smartphone prototype. High-quality fingerprint images were obtained from the sensor on the full-high-definition display of the smartphone, without affecting the display imaging. This study demonstrates the high applicability of the transparent capacitive-type on-screen fingerprint sensors fabricated by using mass-production-ready materials and technologies to smartphones, and other mobile devices. Further studies can be carried out to increase the sensing distance and develop large-area fingerprint-touch combined sensors and sensors for flexible/stretchable devices.

## Materials and methods

### Moiré analysis with pattern images

Grayscale display and sensor pattern images with 16,384 × 16,384 pixels and a resolution of 0.5 μm/pixel were generated for the analyses. In the generation of sensor pattern images and overlapped images, the transmittance of the ITO layer was considered. The transmittance of the ITO area in the whole visible wavelength range was assumed to be 80%, lower than the measured average values of 86% with the substrate effect and ~94% without the substrate effect. The other area was assumed to be 100% transparent to have a higher contrast in the overlapped images and facilitate identification of Moiré patterns. DFT calculations were performed by using MATLAB’s 2D fast Fourier transform function.

### Sensing signal calculation

The fingerprint sensing signals, i.e., the mutual capacitance and capacitance difference values at the sensing nodes, for different sensor patterns were calculated by using the electrostatic modeling capabilities of COMSOL’s AC/DC module. The fingerprint sensor was modeled with a 24 × 44 electrode array, including an active region consisting of a 12 Tx × 32 Rx electrode array and six grounded dummy electrodes on the borders of each side of the active region. A fingerprint template with a constant pitch was included in the model. The mutual capacitance between the Tx and Rx electrodes at each node in the active region was calculated. The materials of each layer in the model and their characteristics are listed in Table 2.

**Table 2:** Thicknesses and dielectric constants of the constituent layers used in the simulation

Layer	Material	Thickness (μm)	Dielectric constant (at 1 MHz, 25 °C)
Ridge	Human tissue	400	50
Valley	Air	59	1
Cover	Schott D263T glass	100	7.7
OCA	3 M CEF series (acrylic)	15	3.65
Insulating/passivation layer	Dongwoo Fine-Chem DNI-LT09	1	3.5
Substrate	Corning Eagle XG glass	700	5.27

### Sensor fabrication

The sensors were fabricated on 8″ glass wafers by sputter deposition along with conventional photolithography and wet etching processes. First, Rx ITO electrodes were deposited to thicknesses of 0.2 μm by sputtering and lithographically patterned. The lower ITO layer was annealed at 250 °C for 1 h in a furnace under a working pressure of 10 mTorr to obtain a sheet resistance of ~10 Ω/sq. before patterning, while the upper Tx ITO layer was annealed at 235 °C because of the processing temperature limit of the organic insulator between the two electrodes. It is slightly better to have Rx over Tx electrodes to obtain a stronger sensing signal. However, we placed Rx below Tx to use the Tx electrodes, which do not send signals at a given time, as ground and an electromagnetic shield. After Rx electrode patterning, the pad metal, Mo, was sputter deposited to a thickness of 0.2 μm and wet etched. The Mo etchant has good selectivity for Mo over ITO, so the ITO can remain virtually intact. The insulator polymer DNI-LT09 (Dongwoo Fine-Chem Co., Ltd.), an acryl polymer with an epoxy unit, was spin-coated to a thickness of 1 μm, patterned, and cured at 235 °C for 1 h for outgassing. After contact holes were created through the insulator layer, Tx ITO with the same thickness as Rx ITO was deposited on the insulator layer, and again annealed and patterned. The polymer used for the insulator layer was again spin-coated to a thickness of 1 μm to form a passivation layer, and patterned to open the pad area and complete the sensor fabrication. The pad metal formation step was skipped when the test patterns for the Moiré evaluation were fabricated.

### Postprocessing and prototype assembly

To obtain the smartphone prototype, the display module was separated from the smartphone, and the cover layer and OCA were removed from the module by using the rework process, in which the display and cover layer were separated by wires passing between them and the remaining adhesive was cleaned. The sensor on a glass substrate was laminated with a 15 μm thick 3 M CEF series OCA and a 100 μm thick cover glass (Schott D263T), while the metal pad area of the sensor remained uncovered. Subsequently, the laminated sensor and OLED panel were bonded with a 3 M post-ultraviolet-curable OCA with a thickness of 200 μm, and any visible air gaps or bubbles at the bonding interfaces were removed by using an autoclave. The metal pads were connected to the FPCB by using anisotropic conductive film (ACF) bonding, and connected to the fingerprint sensing IC. The Hitachi Chemical MF-347HL5 ACF with conductive balls with diameters of 5 μm was used for bonding.
